# Enantiomeric Mixtures in Natural Product Chemistry: Separation and Absolute Configuration Assignment

**DOI:** 10.3390/molecules23020492

**Published:** 2018-02-23

**Authors:** Andrea N. L. Batista, Fernando M. dos Santos, João M. Batista, Quezia B. Cass

**Affiliations:** 1 Department of Chemistry, Federal University of São Carlos—UFSCar, Rod. Washington Luis s/n, km 235, São Carlos, SP 13565-905, Brazil; andrluca@yahoo.com.br (A.N.L.B.); fernandonppn@gmail.com (F.M.d.S.J.); 2 Institute of Science and Technology, Federal University of São Paulo—UNIFESP, R. Talim 330, São José dos Campos, SP 12231-280, Brazil

**Keywords:** polysaccharide-based CSP, chiral chromatography, enantiomeric excess, VCD

## Abstract

Chiral natural product molecules are generally assumed to be biosynthesized in an enantiomerically pure or enriched fashion. Nevertheless, a significant amount of racemates or enantiomerically enriched mixtures has been reported from natural sources. This number is estimated to be even larger since the enantiomeric purity of secondary metabolites is rarely checked in the natural product isolation pipeline. This latter fact may have drastic effects on the evaluation of the biological activity of chiral natural products. A second bottleneck is the determination of their absolute configurations. Despite the widespread use of optical rotation and electronic circular dichroism, most of the stereochemical assignments are based on empirical correlations with similar compounds reported in the literature. As an alternative, the combination of vibrational circular dichroism and quantum chemical calculations has emerged as a powerful and reliable tool for both conformational and configurational analysis of natural products, even for those lacking UV-Vis chromophores. In this review, we aim to provide the reader with a critical overview of the occurrence of enantiomeric mixtures of secondary metabolites in nature as well the best practices for their detection, enantioselective separation using liquid chromatography, and determination of absolute configuration by means of vibrational circular dichroism and density functional theory calculations.

## 1. Introduction

Secondary metabolites play a significant role in drug discovery and development processes [1], as they are intrinsically endowed with both architectural and stereochemical complexity. The possibility of obtaining modified natural products that create diversity of pharmacological properties and improved pharmaceutical results has motivated further advancements in the field [2].

On examining the chiral drug market, Calcaterra and D’Acquarica [3] noticed that all new small-molecule drugs approved by the US Food and Drug Administration (FDA) in 2015 were single enantiomers with defined absolute configuration (AC), with the exception of lesinurad (a racemate of two enantiomeric atropoisomers). They also noted that the chiral-switching practice had not been reported in the 2015 version of the examined dossiers.

**Figure 1:**
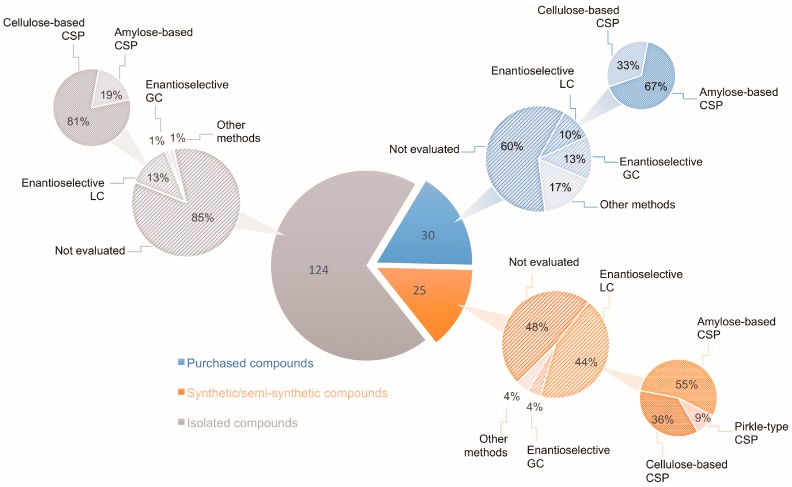
Number of secondary metabolites with AC determined by VOA methods according to Batista et al. [12] and percentage of these compounds for which the enantiomeric composition was evaluated by enantioselective chromatography.

In this context, the search for new chemical entities should always include evaluations of the stereoisomeric composition of the target compounds, when applicable. In the absence of such evidence, the relationship between biological activity and structure may be seriously compromised. Regarding natural products, it is commonly taken for granted that chiral secondary metabolites are biosynthesized in an enantiomerically pure form [4,5,6,7,8,9]. Another factor rarely taken into consideration is the racemization of stereolabile secondary metabolites induced by extraction procedures [10,11]. Thus, although chirality is a key feature of natural products, and stereochemistry is crucial for biological activity, most natural product chemists are predominantly concerned with isolation, biological activity evaluation, and structural elucidation steps, without focusing on the evaluation of enantiomeric composition. This can be envisaged by examining the statistics of isolated chiral secondary metabolites as reported by the *Journal of Natural Products* in 2017. Out of 268 papers describing chiral molecules, only 31 (11.6%) checked the enantiomeric purity of their isolated compounds. Even when the assignment of the AC of a given compound is intended, its enantiomeric composition is generally overlooked. Figure 1 illustrates the data obtained from a recent review [12] covering the stereochemical properties of natural products that were determined using vibrational circular dichroism (VCD) and/or Raman optical activity (ROA). Out of the 124 isolated secondary metabolites reported, only 15% had their enantiomeric ratio determined, mainly by enantioselective chromatography (Figure 1). These results indicate that the enantiomeric composition of many-isolated natural products is still unknown; especially considering the number of natural compounds produced in a non-enantiomerically pure form [13]. Selected examples of naturally-occurring enantiomeric mixtures are discussed herein.

**Figure 2:**
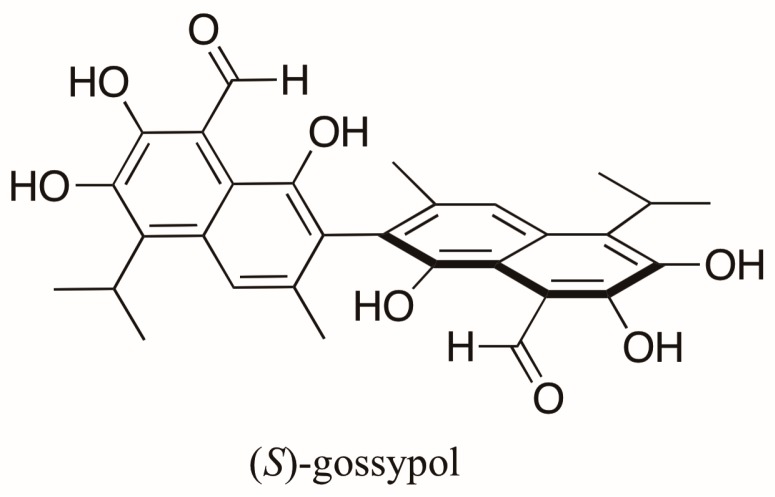
Structure of (*S*)-gossypol, a polyphenolic bissesquiterpene isolated from *Gossypium* species.

Gossypol (Figure 2), an axially chiral and biologically active compound from cottonseed, is a polyphenolic bissesquiterpene isolated as a mixture of (+)- and (−)-enantiomers, which correspond to (*S*)-gossypol (*P* form) and (*R*)-gossypol (*M* form) [14], respectively. This compound has attracted attention because of its antifertility, antioxidant, anticancer effects, among others [15]. Its enantiomers differ in biological activity with (*R*)-gossypol displaying the highest activity [15,16,17]. Interestingly, different *Gossypium* species produce both enantiomers in varying proportions. In varieties of *Gossypium arboreum*, *G. herbaceum* and *G. hirsutum* an enantiomeric excess (*e.e.*) of (*S*)-gossypol ranging from 8 to 90% occurs predominantly, whereas (*R*)-gossypol is present in excess in Brazilian varieties of *G. barbadense* (4–30% *e.e.*). These enantiomeric ratios were measured indirectly by liquid chromatography (LC) after conversion of gossypol to its Schiff’s base diastereoisomers using l-phenylalanine methyl ester [18]. The first direct resolution of racemic gossypol using enantioselective chromatography was carried out almost a decade later [19]. (*R*)-gossypol was found, as expected, in modest excess in the seeds of *G. barbadense* (*Brasiliense tussac*), but (*S*)-gossypol was found in excess in the roots and flowers of different cultivars of this variety and in the seeds of other *Gossypium* species studied [20].

**Figure 3:**
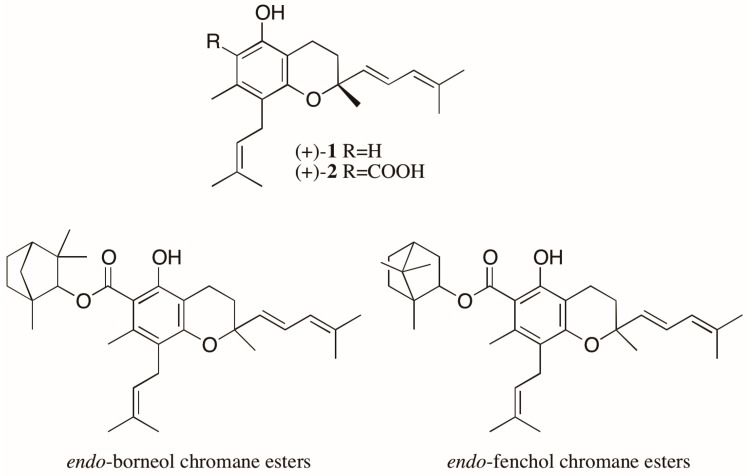
Structure of the chromanes isolated from *Peperomia obtusifolia* as enantiomeric mixtures.

Another noteworthy case involves the trypanocidal prenylated chromanes, peperobtusin A (**1**) and 3,4-dihydro-5-hydroxy-2,7-dimethyl-8-(3′′-methyl-2′′-butenyl)-2-(4′-methyl-1′,3′-pentadienyl)-2*H*-1-benzopyran-6-carboxylic acid (**2**) (Figure 3), isolated as racemates from *Peperomia obtusifolia* (Piperaceae) [21]. From this same plant, eight monoterpene chromane esters were also isolated. Four of them were elucidated as esters of both enantiomers of chromane **2** with both enantiomers of *endo*-borneol, while the other four were characterized as both enantiomers of chromane **2** esterified with both enantiomers of *endo*-fenchol (Figure 3) [22,23]. The biological activities of the stereoisomers of these chromanes were not evaluated, but reports found in the literature show different trypanocidal activity for the enantiomers of a prenylated chromene isolated from another Piperaceae species, *Piper gaudichaudianum*. Interestingly, in this case, mixtures of enantiomers showed a synergistic effect, with the racemate being the most active [24].

A very recent work described six alkaloids isolated from *Chelidonium majus* identified as enantiomeric enriched mixtures by combined analysis of their NMR data, electronic circular dichroism (ECD) spectra, calculations of specific rotations, and enantioselective chromatography profiles [25].

Additionally, eight stereoisomeric 2,3-dihydrobenzo[b]furan neolignans consisting of four diastereoisomers and their four enantiomers were isolated from *Gardenia ternifolia* Schumach. and Thonn. (Rubiaceae) [26]. Their ACs were assigned online by ECD analysis coupled to enantioselective LC. The authors also emphasized the risks of overlooking enantiomeric mixtures in herbal medicines, especially when metabolites biosynthesized through free-radical processes are present.

As exemplified, enantiomeric mixtures of secondary metabolites are not uncommon in Nature and their occurrence is generally underestimated. In the past, natural product chemists used to rely on polarimetry to draw conclusions about either enantiomeric purity or AC of isolated chiral secondary metabolites [27]. Even today, however, it is not unusual to find stereochemical assignments based on comparisons of chiroptical properties (optical rotation, sometimes measured in different conditions, and/or ECD) for structurally correlated compounds [28,29,30], or even based on biosynthetic considerations [31,32]. It is important to mention that herein we are not questioning the referenced assignments, but rather highlighting practices that may result in misassignments.

**Figure 4:**
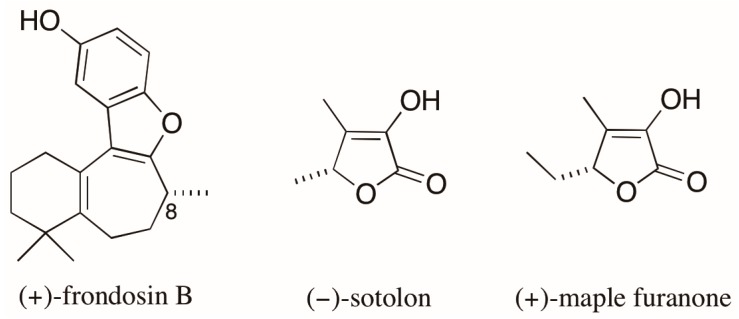
Structures of (+)-frondosin B, (−)-sotolon, and (+)-maple furanone.

An emblematic example of the problems associated with AC assignments based on optical rotation (OR) values involves the case of the marine sesquiterpene frondosin B (Figure 4). Many attempts to assign the AC of the naturally occurring (+)-enantiomer of frondosin B by total synthesis led to conflicting results. The differences in OR obtained for products of distinct synthetic routes had been attributed to an unexpected inversion of the C-8 stereogenic center. However, a recent report by Joyce et al. [33] demonstrated that the presence of a minor impurity (ca. 7%), that appears in a key step late in the synthesis, was responsible for measured OR values of opposite signs for the synthetic products with the same AC. As a result, the assignment of the absolute stereochemistry of the natural product was also compromised [33]. The authors finally state that the reported inversions of the AC assigned at C-8 came “*only from differences observed in the measurements of a small optical rotation conducted on minute quantities of material, often of unknown chemical or enantiopurity*”.

Finally, the case of the of the chiral furanones sotolon and the maple furanone (Figure 4) [34] illustrates the risks arising from assigning AC based on the comparisons of OR values for compounds with similar structures. These 5-substituted-2(5*H*)-furanones differ from each other by the presence of a single methylene unit and, despite having the same AC, they have opposite signs of OR. 

As demonstrated, it is extremely important to measure stereoisomeric composition of natural products and properly determine their AC. Thus, in the next sections the reader will be provided with a tutorial of the best practices for the efficient method development for chiral separations using LC as well as for the determination of AC using VCD and DFT calculations. The following tutorial sections represent general guidelines from the author’s perspective with selected examples and are not intended to exhaustively review the literature.

## 2. Tutorial

**Figure 5:**
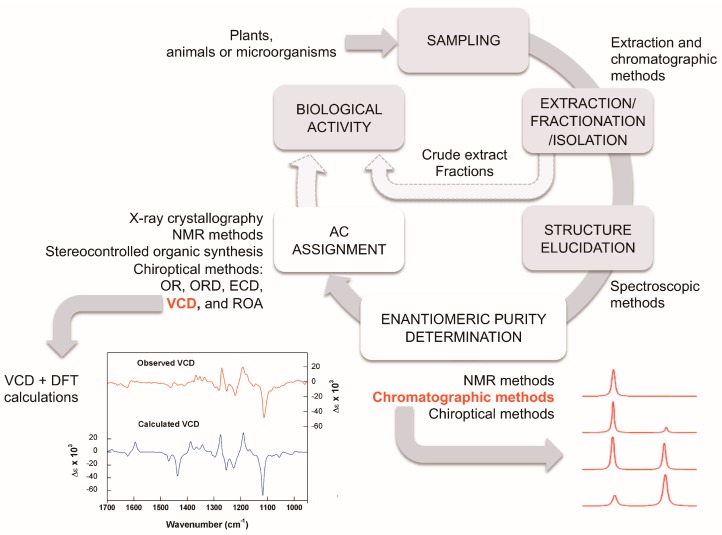
Proposed workflow for the isolation and characterization of natural products. Details of some of the techniques can be found in the main text.

The study of natural products generally involves the following steps: extraction, fractionation, isolation, structural elucidation, and in the case of chiral molecules, enantiomeric purity and AC determinations (Figure 5). In the search for bioactive compounds or bioguided extraction, these steps must be accompanied by biological assays for the evaluation of the desired activity. It is important to emphasize that due to the possible differences in the biological activity of enantiomers and other stereoisomers, the enantiomeric or stereochemical purity of the isolated substances should always be monitored.

Different definitions of enantiomeric proportion are used depending on how the mixture of enantiomers is quantified [35]. Herein, the terms enantiomeric ratio (*e.r.*) and *e.e.* will be employed when non-chiroptical (chromatographic or NMR) and chiroptical methods are used, respectively. However, for published work, the terms are reported as present in the original publications.

### 2.1. Enantioselective Chromatography

Chiral stationaries phases (CSPs) have revolutionized the way that enantiomeric composition is measured; with LC being the most used technique [3,36,37]. CSPs made possible the direct resolution of enantiomers without the need of derivatization, in which enantiomers were converted to diastereoisomers prior to the chromatographic separation (the indirect method). The indirect method has been extensively used even though many drawbacks, such as the lack of enantiomeric purity of the chiral derivatizing agent, could result in false values of enantiomeric composition.

A number of reviews cover the main aspects relating to the type and classification of the chiral selectors used for preparation of the CSPs [37,38,39,40,41,42,43]. The first classification of the chiral selectors was made by Wainer, as early as 1987 [44,45], in accordance with the chiral recognition mechanism. Much later they were classified as synthetic (Pirkle type, ligand exchange, chiral crown ethers, synthetic polymers) and natural selectors (proteins, polysaccharides, cyclodextrins, macrocyclic glycopeptides, cinchona alkaloids, cyclofructans) [41]. Recently, the Wainer classification has been revisited to try to accommodate newly developed CSPs [46] and also to help in the distinction between the different chiral phase groups.

The main focus of enantiomer separation is placed on the CSPs to be used; this can be noticed through the years, since the 1980’s, when the commercial columns became available. The progress in this respect has been amazing and nowadays a wide variety of CSPs are available to be used in different elution modes [37,43,46].

Regarding the molecular bases for the chiral recognition, depending on the selector, the main interactions involved are hydrogen bonding, π–π, dipole–dipole, steric hindrance and inclusion complexes [37,47]. For the formation of the transient diastereoisomers not only does the tridimensional structure play a role in the recognition mechanism, but also the solvated structures of the enantiomers and stationary phases [48].

Unfortunately, there is no universal chiral selector for designing the enantioselective separation of an unknown enantiomeric mixture. Moreover, not only is the chiral selector important but also the support used for preparing the phases, which contributes to the retention behavior by non-stereoselective adsorption [38]. The mode of elution, as well as the organic modifier used, is also of paramount importance in finding the appropriate enantioseparation [37,49].

Hence, finding the CSP at the correct elution mode for measuring *e.r*. is not a trivial task and, in spite of the knowledge that some classes of compounds are well resolved in a given CSP [39,49], the procedure is mainly based on a trial and error approach, and requires systematic search [50,51]. The use of ChirBase, a database that has more than 200,000 records from the literature is helpful [50,51]; but so far, there is no universal predictive model for selecting a CSP for a given enantiomeric mixture [51,52,53].

To meet this end, chiral screening technologies have been pursued using either traditional columns (5 μm fully porous particles) [49,54,55,56,57,58] or multi-column parallel screening [59], as well as other advanced technologies for increasing high throughput and efficiency [60,61]. The ultraviolet (UV) detector is still the most used, but chiral detectors based on polarimetry or ECD has become a tendency for *e.r.* determinations [25,26,62,63]. The use of tandem mass spectrometry for complex sample matrices is also well established [64]. 

#### 2.1.1. The most used CSPs

- Polysaccharide-based CSPs

The amylose and cellulose-based CSPs are by far the most used and are usually the first choice, including their use in supercritical fluid chromatography. They were first introduced back in 1984 [65,66] and since then, a large variety of derivatives of cellulose and amylose coated to silica have been successfully employed [67]. The polysaccharide-based coated phases are used under normal, reverse, or polar organic elution modes [55,68], however modifiers such as acetone, ethyl acetate, dichloromethane, dimethyl sulfoxide and methyl *t*-butyl ether cannot be used due to the solubility of the chiral selectors in these solvents [68,69]. To overcome these problems, despite reducing their chiral recognition ability, polysaccharide-based chiral selectors have been immobilized to silica gel as a gain in technology [48,56,67]. With immobilized polysaccharide-based CSPs the use of a wider number of eluents is possible, which is an important issue for a variety of classes of compounds and, for preparative separation, the solubility of the samples as well as the productivity can be increased [48,70].

Amylose and cellulose offer complementary chiral recognition ability and the *tris*-3,5-dimethyl-phenylcarbamate derivative is the most used in either coated or immobilized forms [67,71]. The separation of the enantiomers of isoborneol, a monoterpenoid, is a fine example of the use of polysaccharide-based CSPs in LC. The enantiomers were separated on semipreparative scale under normal elution mode using an optical rotatory detector, and their ACs were determined by VCD [63]. It is important to mention that chiral monoterpenoids, due to their volatility and few or no functional groups for interaction with the chiral selector, is usually enantionseparated by gas chromatography with cyclodextrin derivatives as chiral selectors [72]. Another interesting example includes the calculation of the enantiomeric ratios of (+)-lyoniresinol in wines, spirits, and wood chip macerates, by LC-HRMS using a Chiralpak^®^ IB-3 column [64]. (+) and (−)-Hyoscyamine have been enantioselectively quantified in *Datura stramonium* and *Brugmansia arborea* seeds, and contaminated buckwheat, using a Chiralpak AY3 column and an LC-QqQ-MS system. By exploring different pH and temperature conditions, the racemization of pure (−)-hyoscyamine to atropine in Solanaceae samples has also been investigated. The racemization occurs rapidly at high temperature and basic pH (pH 9.0). At high temperature (80 °C) and pH 5, the racemization also occurs, but slowly [10]. A Chiralpak^®^ IC was used to enantioselectively separate eight pairs of norlignan, neolignan, and lignan enantiomeric mixtures from *Acorus tatarinowii* with *e.e.* in the order of 66%, 71%, 63%, 60%, 0%, 38%, 48% and 75% [73].

- Macrocyclic antibiotics CSPs

Macrocyclic antibiotics CSPs [74], with vancomycin, ristocetin A, teicoplanin and the teicoplanin aglycone (Chirobiotic^®^) are able to separate diverse classes of compounds. The changes in interaction according to the elution mode make them a wise choice [37]. Xanthones are natural product scaffolds and are very important due to their broad spectrum of biological and pharmacological activities. A series of chiral xanthone derivatives has been enantioseparated, except by one, in the macrocyclic antibiotics CSPs under normal elution conditions. For the separation, the Chirobiotics T, TAG, V and R columns were explored under normal, reversed-phase and polar ionic mode to find the highest resolution for measuring the *e.r*. Docking calculations for the free energy were in agreement with the order of elution of the enantiomers. The calculations also showed that the macrocyclic antibiotic CSPs exhibit binding patterns in accordance with the shape and surface of each chiral selector [57].

Teicoplanin-based chiral stationary phases are well known for their ability to enantionresolve underivatized amino acids [75]. The hydrolysate amino acid constituents of the cyclohexapeptide similanamide, isolated from the marine sponge-associated fungus *Aspergillus similanensis* KUFA 0013, were separated using a Chirobiotic T (teicoplanin) column under reversed-phase elution conditions. The comparison with standard amino acids allowed the elucidation of the isolated cyclohexapeptide as cyclo (anthranilic acid-l-Val-d-Leu-l-Ala-*N*-methyl-l-Leu-d-pipecolic acid) [76].

- Pirkle type CSPs

The evolution of the Pirkle type CSP has been elegantly reviewed [38] and shows the utility of a rational plan for enhancing chiral recognition discrimination [77,78]. The designing of the Whelk-O1 CSP illustrates how the structural features of the chiral selector impact their enantioselectivity. The Whelk-O1 CSP incorporates a donor and an acceptor of π-electrons in a semi-rigid framework that holds a π-acidic 3,5-dinitrobenzamide group perpendicular to a π-polynuclear aromatic group having an amide NH (for hydrogen bond formation) in the cleft formed by the aromatic moieties. This arrangement produces multiple interactions for broader chiral discrimination [38]. The same library of chiral xanthone derivatives already separated using the macrocyclic antibiotic CSPs [57] has been screened using the (*S*,*S*)-Whelk-O1 and l-phenylglycine CSPs under multimodal elution conditions at normal, polar organic, and reversed-phase modes [79]. In this paper, the authors explored not only the elution mode but also the type and concentration of organic modifiers to be able to gain efficiency and shorten the analysis time. Under the screening conditions, three out of seven enantiomeric pairs tested were enantioseparated on the (*S*,*S*)-Whelk-O1 CSP, with α ranging from 1.91 to 7.55 and R_S_ ranging from 6.71 to 24.16. The polar organic elution mode afforded the best resolution at a shorter time [79]. This type of CSP is suitable to be used in the so-called Inverted Chirality Columns Approach for measuring the trace enantiomer in highly enriched enantiomeric mixtures [80,81].

- Cyclodextrin-based CSPs

Cyclodextrins are the only type of CSPs used in gas chromatography [37,40,43]. In this respect, the great ability to separate underivatized enantiomers of varying volatility makes them the preferable CSP for analysis of essential oils [72]. The cyclodextrin-based CSPs have hydrophilic surfaces and hydrophobic cavities enabling them to accommodate diverse classes of compounds by the formation of inclusion complexes. Furthermore, they can be used in normal, reversed-phase or polar organic modes with high efficiency [43]. For an update of the separation mechanisms of these CSPs, especially regarding a series of newly synthetized cyclodextrin derivatives, please refer to the review article by Scriba [37].

As an example of innovative application, the enantiomeric composition of hesperetin in human urine has been determined by LC using a 100 m I.D. capillary column, packed with phenyl-carbamate-propyl-β-cyclodextrin at reversed-phase conditions, in order to evaluate its metabolism and elimination properties [82].

#### 2.1.2. Screening Development

It is acknowledged that the development of efficient conditions for enantiomeric separation should start with the screening of CSPs of complementary chiral discrimination [46,60]. Ideally, a large number of CSPs should be evaluated, what justifies the advancement in screening technology for obtaining separations with high efficiency and throughput [60,61].

Nowadays, a number of LC systems can offer multi-solvent blending and column management options, which allow the use of 6–12 columns (preferable of small size) for automated method development. Supercritical fluid is an excellent approach to find the right conditions for enantiomeric separation. As with LC, it also requires selecting a series of parameters such as CSP and organic modifiers. The new series of SFC systems are able to automatically screen a large number of columns and various mixture ratios of four types of modifiers, which facilitate the efforts required. It should be noticed though, that in day-to-day operations of natural product laboratories these systems are not usual. 

As highlighted by Ahuja [83] “*Cost considerations, availability of equipment, and know-how play important roles in the selection process*”. Accordingly, we should pinpoint some important issues and general recommendations for LC separation of an enantiomeric mixture:

1. Polysaccharide-based CSPs are the ones with the broadest enantiomeric discrimination abilities and are complementary to the macrocyclic antibiotics CSPs. For their screening, it is important to explore multimodal elution, thus, one must be aware of solvent miscibility. 100% ethanol (EtOH) is an excellent choice for changing from normal to polar or reverse-phase modes. As a rule, one should mind the instructions provided by the manufacturer.
2. For the screening of polysaccharide-based CSPs, it is better to start by exploring the normal elution mode with different proportions of alkane (hexane or heptane) to EtOH or 2-propanol. The amylose- and cellulose-*tris*-3,5-dimethylphenylcarbamate derivative CSPs are the ones with the highest success rates [67]. Initially, the polysaccharide coated CSPs were commercialized only by the Daicel; currently, they are commercialized also by other companies with different brand names [71]. With the immobilized CSPs one can explore eluents such as mixtures of alkane with ethyl acetate and tetrahydrofuran (THF) before going to the polar organic mode.
3. The polar organic mode also gives high selectivity and it is usually a good choice for multi-milligram separations. It is important to give time for equilibration, especially for the coated polysaccharide-based CSPs. To avoid immiscibility problems with the eluents, one should go first to 100% EtOH. Methanol (MeOH) or acetonitrile (ACN) are the solvents of choice, or mixtures of them [55,84].
4. Reversed elution mode should also be explored. This is usually done by using acetonitrile or methanol as modifiers in water. The use of buffer solutions is necessary only if the analytes require [19].
5. It is recommended to start the screening with the Chirobiotic^®^ CSPs at polar organic mode for nonionizable enantiomers, or at the polar ionic mode for the ionizable ones. In the first case, ACN is usually used with a small amount of a protic organic solvent, while in the polar ionic mode acid or base is added to MeOH [42]. The manufacturer recommends MeOH with acid and base in proportions 4/1 to 1/4, respectively.
6. The Chirobiotic^®^ CSPs operate well under the reversed mode with MeOH, ACN, and THF as modifiers. The use of an aqueous buffer solution, such as ammonium acetate, is a good selection. The pH can affect drastically the enantioselectivity and should therefore be explored.
7. The normal elution mode is carried out with alkane and 2-propanol or ethanol. The use of acidic or basic additives for Chirobiotic^®^ CSPs under normal elution is also important [57].
8. The use of (*S*,*S*)-Whelk-O1 CSP in the screening should also be incentivized. The highest selectivity is usually achieved under normal elution conditions but it can be explored as well under polar and reversed-phase conditions [38,43,79].

### 2.2. Chiroptical Methods and Absolute Configuration Assignment

Chiroptical methods, which arise from the differential interaction of a chiral non-racemic sample with left- and right-circularly polarized radiation, can also be used for the determination of *e.e.*, since the signal intensity of OR, ECD, VCD, and ROA spectroscopies is proportional to the enantiomeric excess [85,86,87,88,89,90]. This property of chiroptical methods enables direct measurement of the percentage of a given enantiomer without previous separation [90]. VCD can discern up to 1% differences of *e.e.*, while ECD has its discerning power within the range of 1–0.1% for samples with high CD magnitudes. OR, and its wavelength dependent counterpart, optical rotatory dispersion (ORD), discern 0.1% *e.e.* differences or better for samples with high specific rotations [91]. Although chiroptical spectroscopy can be used for the determination of *e.e.*, this should not be performed based on single measurements, even if this is a common practice in natural product chemistry. In reporting the isolation of eight enantiomeric mixtures from *Acorus tatarinowii*, the authors call attention to the fact that many enantiomeric mixtures are inadvertently considered as enantiomerically pure molecular entities due to the obtained optical rotations and/or Cotton effects in their ECD spectra [73].

Instead, it is recommended that calibration curves be constructed to detect a given compound in the presence of different concentrations of its enantiomer. Single measurements of any chiroptical property may lead to erroneous conclusions regarding enantiomeric purity and, possibly, AC. 

The determination of ACs can be achieved using several methods, including X-ray crystallography, NMR methods, chemical correlation method, and stereocontrolled total organic synthesis. Even though X-ray crystallography was used in the first assignment of the AC of a chiral compound [92], and is still considered the most reliable technique, it requires a well-defined single crystal and the presence of at least one strong anomalous scatterer (a heavy atom). In the past, in the absence of such a scatterer, an internal chiral reference of known AC had to be introduced into the crystal structure [93,94]. Recent progress, especially the use of Cu *k*α radiation, has enabled confident assignment of absolute structure even for hydrocarbons [95]. While very powerful for structure determination, NMR spectroscopy is intrinsically achiral in isotropic media, and can be used only if a chiral auxiliary is added during the experiment. NMR auxiliaries may include chiral derivatizing agents, chiral solvation agents, ion-paring agents, chiral hosting compounds, metal complexes, and liquid crystals [96]. Finally, stereocontrolled organic synthesis is usually laborious, time-consuming, expensive [97], and highly dependent on the correct AC of both starting materials and products.

As an alternative, chiroptical methods have faced a renewed interest in their use for determining molecular stereochemistry [98]. This ever-growing interest in chiroptical properties results, among other things, from developments in *ab initio* [99] calculations for predicting theoretical spectra implemented in user-friendly software programs, and the availability of commercial instrumentation. In this way, the comparison between calculated and observed data greatly assists the correct interpretation of experimental information. These methods are non-destructive and can be measured directly in solution, without the need for crystallization. The power of chiroptical spectroscopy for the stereochemical characterization of organic compounds is based on the fact that the two mirror-image circularly-polarized light beams interacting with a chiral molecule is a manifestation of diastereomeric discrimination [100].

Following Nafie’s proposal at the Pharmacopeial Forum in 2013 [101], the United States Pharmacopeia has recently included the use of VCD as a tool for AC assignment, as well as the determination of enantiomeric purity, of chiral pharmaceutical ingredients at all stages of the discovery process [3]. VCD combines the wealth of structural information inherent to IR spectroscopy with the sensitivity to chirality common to all chiroptical methods. The complete analysis of IR/VCD spectra provides not only information about absolute configuration, but also the conformational population in solution.

Despite the advances and advantages described above, as well as the steady growth in the number of secondary metabolites studied by VOA methods in the last decades [12,102], VCD is not widely used in the determination of the AC of natural products. One of the reasons for this observation is that VCD is still considered a spectroscopic novelty and remains unknown to many researchers in the field. Furthermore, the need of quantum chemical calculations in order to interpret experimental data, in most cases, precludes its use on a daily basis by non-experts. In the following sections, general recommendations for measurements and calculations of quality IR and VCD spectra will be presented.

#### 2.2.1. VCD Measurements

Among all the methods used to determine the AC of chiral molecules, VCD appears as an excellent tool for the analysis of chiral natural products. This technique has many advantages over other methods widely used since there is no need of single crystals, derivatizations, or ultraviolet-visible (UV-Vis) chromophores. Although non-empirical methods [103] as well as spectra-structure relationships [104] have been developed that do not require theoretical calculations, the most common procedure for AC determination using VCD involves the comparison of experimental data with DFT-predicted spectra for an arbitrarily chosen absolute configuration.

The first steps for the measurement of good quality IR and VCD spectra include the selection of solvent, sample concentration, and path length necessary to obtain an IR spectrum in the mid-IR region (ca. 800–2000 cm^−1^), with an optimum absorbance (A) of approximately 0.5. Absorbance values should neither be lower than 0.1 nor higher than 1.0 absorption units. These conditions may require that different regions of the spectrum be measured separately to achieve an appropriate level of absorbance that results in a reliable VCD spectrum. The reliability of a VCD measurement is often assessed by checking the VCD noise level spectrum.

As the differential response of a chiral molecule to circularly-polarized light is proportional to the ratio of the size of the vibrational chromophore and the wavelength of the radiation, the phenomenon of CD in the infrared region is ca. 10 to 100 times weaker than that in the UV-Vis [97]. As a result, the measurement of a quality VCD spectrum requires larger amounts of sample and longer collection times when compared to ECD. Generally, concentrations of 0.01–0.1 M are required. Sample cells of different volumes (~50–200 μL); different path lengths (~5–10 μm for aqueous samples; ~50–200 μm for non-aqueous samples), and of either CaF_2_ or BaF_2_ windows are the most commonly used. The former transmits to 1200 cm^−1^, while the latter provides coverage down to 800 cm^−1^. To minimize the influence of vibrational transitions of the solvent in the measured VCD spectrum, either deuterated solvents, such as D_2_O, DMSO-*d*_6_, methanol-*d*_4_ and CDCl_3_, or those devoid of C–C bonds, such as CCl_4_ and CS_2_, are commonly used.

IR, VCD, and noise spectra are usually collected with a spectral resolution in the range of 4–8 cm^−1^ by using blocks of variable time length, which are then averaged to yield the final spectra. The total collection time of a VCD spectrum varies roughly from 1 to 12 h, and the signal-to-noise ratio is proportional to the square root of the number of blocks. Once a VCD spectrum is measured, its baseline must be corrected either by subtracting the VCD spectra of both enantiomers and dividing by two (best option) or by subtracting the VCD spectrum of the corresponding racemate. In case neither of the above is available, subtraction of the VCD spectrum of the solvent must be carried out, with the best results being obtained in this case using a dual-PEM system.

#### 2.2.2. Calculations of Theoretical VCD Spectra

IR/VCD spectral calculations include initially the construction of a three-dimensional input structure, whose AC is arbitrarily chosen and based on the relative configuration determined from NMR and/or X-ray experimental data. Subsequently, a thorough conformational analysis using molecular mechanics, molecular dynamics or semi-empirical methods, is carried out. The conformers identified are filtered out according to an energy criterion (relative energy ~5–10 kcal/mol), and subjected to further geometry optimization steps using DFT (commonly B3LYP/6-31G(d)). Dipole and rotational strengths, which are proportional to IR and VCD intensities, respectively, are then calculated at the same level of theory used for geometry optimization. The set of dipole or rotational strengths are finally converted to a full IR or VCD spectrum to be compared with experiment generally assuming Lorentzian band shapes. 

After the IR and VCD spectra of the contributing conformers have been calculated, they are weighted by their fractional Boltzmann population and summed to produce the final calculated IR and VCD spectra. For best comparison with the experiment, the calculated frequencies are uniformly scaled to compensate for the anharmonicity of the observed frequencies. For DFT (B3LYP/6-31G(d)), a scale factor of 0.97 is typically used for the frequencies, and a bandwidth of 6 cm^−1^ is considered for comparison with experimental spectra measured at 4 cm^−1^ resolution. Higher levels of theory, such as B3PW91/cc-pVTZ, may require a scale factor of 0.98 [105]. Both observed and calculated spectra are preferentially plotted in stack mode with VCD above the IR on the same wavenumber frequency scale. Plotting in this manner allows visual correlation between IR peak features and VCD peak features.

Currently, the method for calculating VCD is available in commercial software such as GAUSSIAN 16, TURBOMOLE 7.2, among others. Most VCD calculations are carried out at the DFT level using the hybrid functionals B3LYP or B3PW91 combined with the 6-31G(d) basis set, considered the minimum basis set for VCD. At least for small molecules, a larger basis set such as cc-pVDZ, cc-pVTZ, TZ2P, and TZVP provide better results compared with 6-31G(d). For larger molecules, however, it has been found that the triple basis set TZVP does not provide practical advantages over 6-31G(d) as far as the accuracy-to-time ratio is concerned [22]. When choosing a basis set for VCD calculations, one has to consider that polarization functions are necessary, while diffuse functions do not seem to improve significantly the results. 

Solvent effects on vibrational rotational strengths are typically small, especially for non-polar solvents. However, solvent effects are supposed to affect the optimized geometries and the conformer population in solution. These effects can be accounted for by using different approaches, such as the inclusion of the polarizable continuum model (PCM), the conductor-like screening model (COSMO), or even the inclusion of explicit solvent molecules. Explicit solvation is particularly useful when solute-solvent interactions influence both the conformational population and frequencies/intensities of certain vibrational modes [106]. Inclusion of explicit molecules may also be necessary to correctly reproduce the formation of molecular aggregates, such as carboxylic acid dimers, which are observed for concentrated solutions in apolar solvents [106,107].

As experimental IR and VCD data are commonly recorded in deuterated solvents, isotopic exchange is also expected to occur when hydroxylated molecules are analyzed in protic solvents [108]. Therefore, in the case of stereochemical analysis of polar chiral natural product molecules, a combination of VCD and ECD is recommended. As ECD probes electronic transitions of a given molecule and does not require the use of deuterated solvents, it is not subjected to isotopic effects [108].

Finally, when the comparison between calculated and observed data is performed to assign AC, the visual correlation may not be enough for an unambiguous assignment. To assess the level of agreement between calculated and measured VCD spectra, some approaches can be used. The first one consists of extracting dipole and rotational strengths from experimental data and plotting it against the calculated ones. This method offers the possibility of calculating statistical measures, such as the correlation coefficient R^2^ [109]. A second method called SimIR/ VCD [110] uses computationally optimized frequency scaling and shifting to match calculated and observed spectra. A third method is the confidence level algorithm [111] that provides a direct quantitative comparison of experimental and calculated spectra for both enantiomers as a measure of the degree of agreement and hence level of confidence. One of the most recent methods is based on the similarity of dissymmetry factor spectra placing emphasis on robust regions both in the experimental and calculated spectra [112].

## 3. Conclusions

Chirality is ubiquitous in Nature and it is reflected, at molecular level, in the fascinating structural complexity of many secondary metabolites. As demonstrated in this review, however, not always is the same enantiomer/stereoisomer biosynthesized by a given species nor is it produced necessarily in an enantiomerically pure/enriched fashion. These latter facts are commonly overlooked by natural product chemists, even when they attempt to determine the AC of the isolated compounds. A common protocol to determine enantiomeric purity and, sometimes AC, of naturally occurring compounds still involve OR measurements. As discussed in the literature [26], the purification of enantiomers has been reported mostly for true racemates (null ECD or specific rotation). For enantiomerically enriched mixtures of natural products, the measured chiroptical properties, which are dictated by the enantiomer in excess, are frequently reported without information of their enantiomeric proportion [73]. Based on the risks associated with relying on stereochemical information derived from OR data, herein, we presented the main guidelines for reliable and unambiguous determinations of both enantiomeric purity and AC of chiral secondary metabolites. The combination of enantioselective chromatography and VCD spectroscopy is then recommended as a powerful protocol to be incorporated into the natural product chemistry toolbox.

## Glossary

In this section we will present definitions of some of terms used in the text based on the IUPAC Compendium of Chemical Terminology (Gold Book) [113]:

*Enantiomer excess (enantiomeric excess)*: For a mixture of (+)- and (−)-enantiomers, with composition given as the mole or weight fractions *F*_(+)_ and *F*_(−)_ (where *F*_(+)_ + *F*_(−)_ = 1) the enantiomer excess is defined as | *F*_(+)_ − *F*_(−)_ | (and the percent enantiomer excess by 100 | *F*_(+)_ − *F*_(−)_ |). Frequently this term is abbreviated as *e.e.*
*Enantiomeric purity*: See: enantiomer excess
*Enantiomeric ratio*: The ratio of the percentage of one enantiomer in a mixture to that of the other e.g., 70 (+):30 (−).
*Enantiomerically enriched*: A sample of a chiral substance whose enantiomeric ratio is greater than 50:50 but less than 100:0.
*Enantiomerically pure*: A sample for which all of its molecules have (within the limits of detection) the same chirality sense. Use of homochiral as a synonym is strongly discouraged.
*Racemate*: An equimolar mixture of a pair of enantiomers. It does not exhibit optical activity. The chemical name or formula of a racemate is distinguished from those of the enantiomers by the prefix (±)- or *rac*- (or *racem*-) or by the symbols *RS* and *SR*.
